# Chromatin Immunoprecipitation dataset of H3ac and H3K27me3 histone marks followed by DNA sequencing of *Medicago truncatula* embryos during control and heat stress conditions to decipher epigenetic regulation of desiccation tolerance acquisition

**DOI:** 10.1016/j.dib.2022.107793

**Published:** 2022-01-05

**Authors:** Jaiana Malabarba, Zhijuan Chen, David Windels, Jerome Verdier

**Affiliations:** University Angers, Institute Agro, INRAE, IRHS^1^, SFR QUASAV, Angers F-49000, France

**Keywords:** ChIPseq, H3K27me3, H3ac, Seed maturation, Desiccation tolerance, Heat stress, Embryo

## Abstract

Desiccation tolerance (DT) is one of the most important processes that seeds need to acquire during seed maturation because it will ensure survival until seeds have favourable conditions for germinating. Moreover, in the current climate warming context, heat stress and its impact on seed maturation and quality has been increasingly studied by the scientific community. Even if the transcriptomic changes enrolled in DT acquisition and seed heat stress response are fairly known, its epigenetic control has not yet been investigated. *Medicago truncatula* is a model legume for studying seed molecular mechanisms, which is known to display a delay in the acquisition of seed maturation mechanisms under heat conditions, except for desiccation acquisition. Our aim was to evaluate the role of two histone marks during embryo development under control and heat stress conditions on seed maturation processes, including the DT acquisition. These histone marks have either repressive (H3K27me3) or inducible (H3ac) effects on gene transcription, respectively corresponding to markers of packed and accessible chromatins. We identified all genomic regions bound to the H3K27me3 histones at four developmental stages and to the H3ac histones at the two earlier developmental stages during seed maturation, from seed filling to mature dry seeds, collected under optimal and heat stress conditions in the model legume, *Medicago truncatula* (reference genotype A17). A list of genes and promoters potentially linked to these two histone marks is reported and could provide clues about the epigenetic regulation of seed maturation between control and heat stress conditions, including the desiccation tolerance acquisition.

## Specifications Table

**Table:** 

Subject	Agricultural and Biological Sciences
Specific subject area	Omics: Epigenetics Plant Science: Plant Physiology
Type of data	Tables Figures
How data were acquired	Libraries were constructed following MicroPlex Library Preparation Kit v2 High Performance Library Preparation for Illumina® NGS Platforms (Diagenode SA. Liège, Belgium). DNA libraries samples were sent to BGI, Hong Kong, for sequencing. Samples contained on average 100 ng/µl of DNA. Sequencing resulted in paired-end 100 nucleotide reads (PE100).
Data format	Filtered raw reads (FASTQ) Analysed ChIP-seq data files (peak calling BED files) Metadata Available at https://www.ncbi.nlm.nih.gov/geo/query/acc.cgi?acc=GSE184439
Parameters for data collection	Chromatin immunoprecipitation assays were performed on embryos dissected from four seed developmental stages of *Medicago truncatula* (A17) seeds that were harvested during maturation phase before and after acquisition of desiccation tolerance (respectively S1 and S2) and at the onset and after longevity acquisition (respectively S3 and S4) under standard temperature (20°C day/ 18°C night) and under high temperature (26°C/24°C). Monoclonal antibodies for histone markers H3K27me3 and H3ac (pan-acetyl) were added to purified crosslinked chromatin at 3µg of antibody per 250 µl of ChIP dilution buffers plus purification beads. Antibody and histone binding occurred overnight at 4°C under agitation, prior to magnetic bead purification then decrosslinking.
Description of data collection	ChIP-seq dataset were collected from paired-end sequencing of DNA libraries using BGISeq500 platform with 100bp reads. Raw reads were filtered to remove adapters and low-quality reads, then mapped to *Medicago truncatula* reference transcriptome (version 5). Mapping was performed using STAR mapper, read deduplication using Picard and peak calling using macs2 callpeak. Samtools algorithm was used to generate bigwig. From peak calling, genes were annotated based on gene coding region and in 3Kb promoter regions by bedtools intersect.
Data source location	Controlled growth chambers from the Institut de Recherche en Horticulture et Semences (IRHS), INRAE City: Beaucouzé Country: France
Data accessibility	Public Repository: Repository name: NCBI GEO Data identification number: GSE184439 Direct URL to data: https://www.ncbi.nlm.nih.gov/geo/query/acc.cgi?acc=GSE184439

## Value of the Data

• Our data represents a valuable and unique dataset of seed chromatin immunoprecipitation of histone mark H3K27me3 and H3ac (pan-acetyl) during the seed development in embryo of *Medicago truncatula* upon control and heat stress conditions.
• These data can be exploited by the scientific community as useful resources to investigate the change of the repressive H3K27me3 and the active H3ac histone marks upon (i) the embryo development in control conditions, (ii) the embryo development during heat stress conditions and (iii) the acquisition of desiccation tolerance (between S2, desiccation insensitive stage and S1 desiccation sensitive stage)
• Furthermore, we make available our ChIP-seq protocol (chromatin immunoprecipitation/purification protocol and bioinformatic pipeline), which can be useful as a template for any ChIP-seq data analysis.

## 1 Data Description

**Fig. 1:**
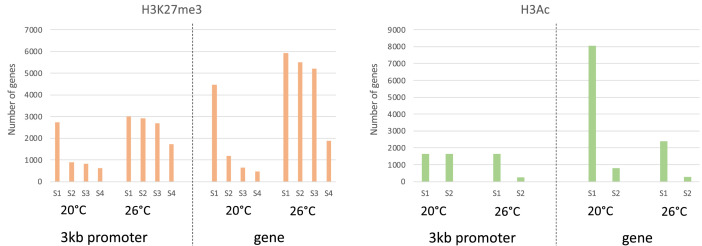
Number of genic regions (*i.e.* 3kb promoter and/or coding sequences) immuno-precipitated with either H3K27me3 or H3ac during developmental stages of *M. truncatula* embryos.

This manuscript presents data sets produced by chromatin immunoprecipitation followed by DNA sequencing of two histone markers (H3K27me3 and H3ac) derived from *Medicago truncatula* embryo. Seed samples are from four developmental stages of *M. truncatula* (A17) embryos during maturation phase [*i.e.* before and after desiccation tolerance (respectively S1 and S2) and at the onset and after longevity acquisition (respectively S3 and S4)] produced under standard temperature (20°C day/ 18°C night) and heat stress (26°C/24°C). Heat stress conditions (26°C/24°C) were chosen based on the recommended Medicago growth conditions available in the Medicago handbook (https://www.noble.org/medicago-handbook/). Indeed, despite to be a Mediterranean species, *Medicago truncatula* behaves as winter annuals with germination in autumn and flowering time early spring. Table 1 displays all sample names with information regarding histone markers, treatments, developmental stages and numbers of reads sequenced per sample. **File S2** provides sequence quality histograms obtained from FastQC [1] and merged into a single graphic using MultiQC [2]. All 24 samples displayed Phred quality scores about 35, which corresponds to a base calling accuracy of about 99.95%. The entire script used to analyze these data is provided as Supplementary **file S1**. In summary, STAR algorithm [3] was used to map paired-end reads to the *Medicago truncatula* reference genome (**File S3**) [4]. Deduplicated reads were marked using Picard algorithm and removed using Samtools [5]. Macs2 [6] was, then, used to call peaks bound to histone marks using the INPUT and IP files, and these peaks were annotated using Bedtools Intersect [7]. Lists of genes that were identified as linked to these histone marks in their 3kb promoters and/or coding sequences are provided in Files S4 and S5. Fig. 1 represents the number of genes (*i.e.* promoter and coding sequence) that were identified as linked to one of these two histone marks at the different stages of embryo development.

**Table 1:** Summary of sample files with corresponding information related to analysed histone marks, seed developmental stages, growth conditions and numbers of clean sequenced reads used for ChIP-seq data analysis.

Embryo stage	Histone mark	Number of reads	Growth (°C)	Input or IP sample
S1	H3K27me3	90.8 M	20°C	IP
S1	H3K27me3	91.1 M	**26°C**	IP
S2	H3K27me3	120.4 M	20°C	IP
S2	H3K27me3	92.6 M	**26°C**	IP
S3	H3K27me3	101.2 M	20°C	IP
S3	H3K27me3	122.4 M	**26°C**	IP
S4	H3K27me3	77.3 M	20°C	IP
S4	H3K27me3	81.3 M	**26°C**	IP
S1	H3K27me3	139 M	20°C	INPUT
S1	H3K27me3	120.6 M	**26°C**	INPUT
S2	H3K27me3	125.3 M	20°C	INPUT
S2	H3K27me3	158.5 M	**26°C**	INPUT
S3	H3K27me3	125.8 M	20°C	INPUT
S3	H3K27me3	129 M	**26°C**	INPUT
S4	H3K27me3	205.3 M	20°C	INPUT
S4	H3K27me3	133.5 M	**26°C**	INPUT
S1	H3ac	18 M	20°C	IP
S1	H3ac	21.6 M	**26°C**	IP
S2	H3ac	22.5 M	20°C	IP
S2	H3ac	34 M	**26°C**	IP
S1	H3ac	95.7 M	20°C	INPUT
S1	H3ac	18.7 M	**26°C**	INPUT
S2	H3ac	31.4 M	20°C	INPUT
S2	H3ac	25.4 M	**26°C**	INPUT

## 2 Experimental Design, Materials and Methods

### 2.1 Plant growth conditions and seed sampling

Medicago plants were grown under standard conditions (20°C/18°C, 16h photoperiod) in growth chambers. At flowering time, half of plants were kept at same optimal conditions (20°C/18°C, 16h photoperiod) and half were grown under heat stress conditions (26°C/24°C, 16h photoperiod). Developing and mature seeds were collected from standard and stress conditions, embryo tissues were quickly dissected then immediately frozen in liquid nitrogen. According to our previous study, four seed developmental stages were collected under standard conditions at 17 days after pollination (DAP), 26 DAP, 36DAP and 44 DAP and under heat stress conditions at corresponding developmental stages at 14 DAP, 17 DAP, 22 DAP and 28 DAP. These four developmental stages were shown to be the beginning of seed maturation and before acquisition of desiccation tolerance (S1), after the acquisition of desiccation tolerance and during seed filling (S2), at the onset of the acquisition of longevity and at the end of seed filling (S3) and mature seed (S4) [8].

### 2.2 Chromatin immunoprecipitation, purification and library generation

Chromatin immunoprecipitation and purification protocols were modified from Cortijo et al. (2018) [9] (that was initially modified from Saleh et al. [10]) and adapted to seed tissues. Fifty seeds at early stage (S1 and S2) and 20-25 seeds at the late stage (S3 and S4) were harvested and grinded into fine powder. 20 ml of crosslink buffer (0.4 M sucrose, 10 mM Tris-HCl pH 8, 1 mM EDTA, 1% formaldehyde, Cocktail protease inhibitor) was added to each sample and samples were incubated at room temperature for 10 min. 2 ml of Glycine 2M (freshly prepared) was added and incubated for 5 min to stop the reaction. Crosslink buffer was removed by centrifugation (5 min at 3000 g). Nuclei isolation buffer (0,25 M sucrose, 15 mM PIPES pH 6.8, 5 mM MgCl2, 60 mM KCl, 15 mM NaCl, 1 mM CaCl2, 0.9% Triton-X100, Cocktail protease inhibitor, freshly prepared and kept at 4°C) was added to samples and incubated on ice for 30 min. After, samples were filtered first on 70 µm then 40 µm filters and then centrifuged at 11000g for 20 min at 4°C. Pellets were resuspended in 1ml of Nuclei lysis buffer (50 mM HEPES pH 7.5, 150 mM NaCl, 1 mM EDTA, 1% SDS, 0.1% sodium deoxycholate, 1% Triton X-100, Cocktail protease inhibitor, freshly prepared and kept at 4°C). Samples were then sonicated for 15 min with pulses of 75W (Sonicator Covaris M220). Samples were, then, centrifuged (13.800g for 10 min at 4°C) and supernatants were collected. 100µl of the supernatant was stored at -80°C to serve as INPUT samples, while 600 µl were used for IP samples. IP samples were diluted 10 times with nuclei lysis buffer without SDS. Samples were incubated with 10 µl of magnetic beads (5µl Dynabeads® Protein G/5µl Dynabeads® Protein A) for 2 h at 4°C with gentle agitation. Later on, 20µl of magnetic beads (washed) were added per IP samples (10µl Dynabeads® Protein G/10µl Dynabeads® Protein A) and 3ug of antibody was added to 250 µl of different samples. Samples were then incubated overnight at 4°C with gentle agitation for immuno-binding of H3K27me3 and H3ac (pan-acetyl) monoclonal antibodies (Active Motif, Carlsbad, California, United States). Sample purification followed multiple washes with magnetic beads and the following buffers: low-salt washing buffer (150 mM NaCl, 20 mM Tris-HCl pH 8.0, 0.2% SDS, 0.5% Triton-X100 and 2 mM EDTA); high-salt washing buffer (500 mM NaCl, 20 mM Tris-HCl pH 8.0, 0.2% SDS, 0.5% Triton-X100 and 2 mM EDTA); LiCl washing buffer (0.25 M LiCl, 1% sodium deoxycholate, 10 mM Tris-HCl pH 8, 1% NP-40 and 1 mM EDTA) and TE buffer (1 mM EDTA and 10 mM Tris-HCl pH 8). Samples were resuspended in elution buffer at 65°C (0.5% SDS and 0.1 M NaHCO_3_). Decrosslinking was performed on IP and INPUT samples by adding 0.5 µl 5M NaCl per 10µl of sample and incubated at 65°C overnight. Samples were purified by adding 10 µl of 0.5 M EDTA, 20 µl of Tris-HCl pH 6.5, 1 µl of RNase A and incubating for 30 min at 42°C then adding 1µl protease K (20 mg/ml) and incubated for 1.5 h, 45°C to digest proteins. Equilibrated AMPure beads (Beckman Coulter, #A63880, CA, USA) were used for sample washing (2 (beads): 1 (sample) ratio). Finally, beads were resuspended in 25 µl of Tris-HCl 10mM pH8 and supernatant was used for library preparation. DNA concentration was measured by Qubit™ dsDNA HS Assay Kit (Thermo Fisher Scientific, Waltham, Massachusetts, United States) and 50 ng of purified chromatin were used as starting amount for PCR amplification. Libraries were constructed following MicroPlex Library Preparation Kit v2 High Performance Library Preparation for Illumina® NGS Platforms (Diagenode SA. Liège, Belgium). Library products were evaluated by Qubit and fragment size were measured using a Bioanalyzer 2100 instrument (Agilent Technologies, Santa Clara, CA, USA). Samples were sent to Beijing Genomics Institute (https://www.bgi.com) (Hong Kong) for sequencing on BGISEQ-500 platform, generating at least 40M reads of 100bp per sample.

### 2.3 Bioinformatic analyses of sequenced reads

Raw reads were filtered to remove adapters and low-quality reads, then mapped to *Medicago truncatula* reference transcriptome (version 5) and quality control was performed using FastQC and MultiQC algorithms [1,2] (File S2). STAR algorithm [3] was used to map paired-end reads to the *Medicago truncatula* reference genome (File S2) [4]. Deduplicated reads were marked using Picard MarkDuplicates (https://gatk.broadinstitute.org/hc/en-us/articles/360037052812-MarkDuplicates-Picard) and removed using Samtools [5]. Macs2 callpeak function [6] was, then, used to call peaks bound to histone marks using the INPUT and IP files, and these peaks were annotated using Bedtools Intersect [7] as being located in 3kb promoter regions and/or gene coding regions based on genome annotation. The entire script containing all options/settings used to analyse these ChIPseq dataset is provided in File S1.

## Ethics Statement

This work does not contain any studies with human or animal subjects.

## CRediT authorship contribution statement

**Jaiana Malabarba:** Investigation, Methodology, Visualization, Writing – original draft. **Zhijuan Chen:** Methodology. **David Windels:** Methodology. **Jerome Verdier:** Methodology, Supervision, Conceptualization, Data curation, Writing – review & editing.

## Declaration of Competing Interest

The authors declare that they have no known competing financial interests or personal relationships which have or could be perceived to have influenced the work reported in this article.

## Appendix Supplementary materials

**File S1**: Script used for ChIPseq data analysis Image, application 1

**File S2**: Overview of the Phred quality values across all bases at each position in the fastq files from the 24 samples obtained from FastQC and MultiQC analyses. Image, application 2

**File S3**: Reference genome, from *Medicago truncatula* genome version 5, used in this study and available at https://medicago.toulouse.inra.fr/MtrunA17r5.0-ANR/downloads/1.7/MtrunA17r5.0-ANR-EGN-r1.7.fastaFiles.zip Image, application 3

**File S4**: List of annotated genes (3kb promoter + coding sequences) immuno-precipitated with H3K27me3. Image, application 4

**File S5**: List of annotated genes (3kb promoter + coding sequences) immuno-precipitated with H3ac ChIP-seq. Image, application 5
