# Supplementary figures and images for: Chromatin Immunoprecipitation dataset of H3ac and H3K27me3 histone marks followed by DNA sequencing of Medicago truncatula embryos during control and heat stress conditions to decipher epigenetic regulation of desiccation tolerance acquisition
Source: Data Brief. 2022 Jan 5;40:107793. doi: 10.1016/j.dib.2022.107793 (PMC8749208; doi:10.1016/j.dib.2022.107793)

File S2

## FastQC: Mean Quality Scores

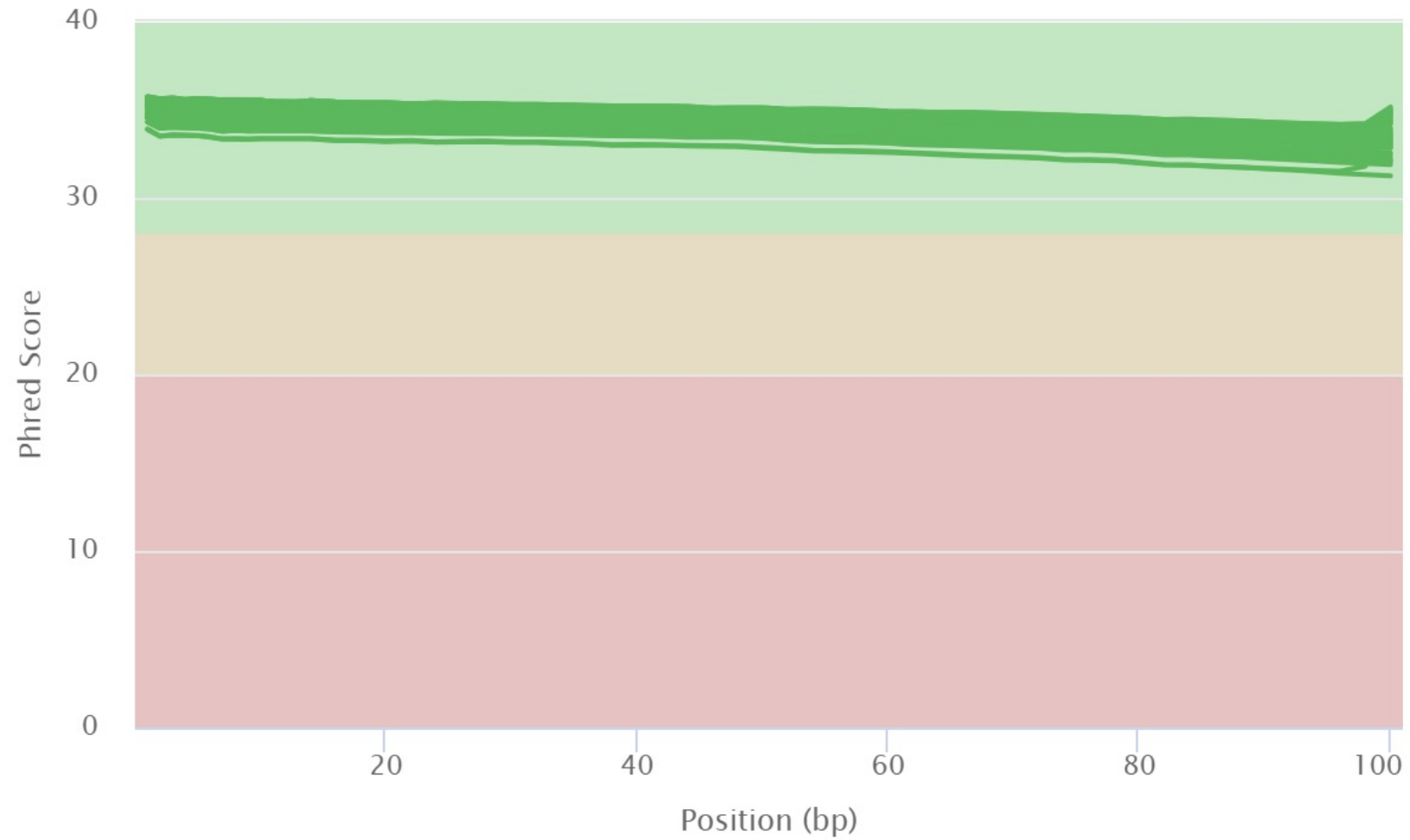

Created with MultiQC

Supplement: Supplementary file 2 — File S2: Overview of the Phred quality values across all bases at each position in the fastq files from the 24 samples obtained from FastQC and MultiQC analyses. [file mmc2.pdf]
